# Can promoting compassion and gratitude through a four-week online training program improve women's mental health? A randomized controlled trial

**DOI:** 10.1186/s12905-025-03763-7

**Published:** 2025-07-22

**Authors:** Lotte Bock, Madiha Rana, Tahnee Rössler, Majeed Rana

**Affiliations:** 1 Department of Psychology, Leu¬Pha¬Na Uni¬Ver¬Sität Lüne¬Burg, Lüneburg, Germany; 2 Department of Health Psychology Europäische Fernhochschule Hamburg, University of Applied Sciences, Hamburg, Germany; 3 Department of Health Psychology Europäische Fernhochschule Hamburg, University of Applied Sciences, Hamburg, Germany; 4 Department of Oral and Maxillofacial Surgery, Heinrich Heine University Duesseldorf, Duesseldorf, Germany

**Keywords:** Compassion, Gratitude, Self-instructed training, Positive psychology, Mindfulness

## Abstract

**Background:**

The period following the pandemic has witnessed a surge in depression, distress, and anxiety, alongside a rise in digitalization. This has underscored the necessity of finding alternatives to in-person interventions for mental well-being. According to positive psychology, compassion and gratitude can alleviate anxiety and depression. This pilot study investigates the impact of a four-week self-directed online training program that emphasizes compassion and gratitude as essential components of women's psychological well-being.

**Methods:**

For this randomized controlled trial, a sample of 51 women aged between 21 and 39 years was selected. The experimental group (*n* = 26) underwent a four-week training program on compassion and gratitude, which included psychoeducation, compassion exercises, and journaling. The control group was a waitlist control group (*n* = 25). Participants' levels of compassion and gratitude were assessed before and after the four-week program using standardized self-report surveys. The German Self-Compassion Scale (SCS-G) and the German Multi-Component Gratitude Measure (MCGM-G) were utilized to examine the differences between the experimental group and the waitlist control group over time, a repeated measures ANOVA was conducted.

**Results:**

The study shows that participants in the experimental group experienced a significant improvement in both compassion and gratitude skills. Furthermore, there was a strong positive correlation between compassion and gratitude.

**Conclusion:**

The findings of the pilot study suggest that a brief self-directed online program aimed at cultivating compassion and gratitude can enhance factors that are crucial to women's mental well-being. Further research is necessary to examine the long-term effects of these interventions and their suitability for diverse demographics.

**Trial registration:**

The trail was registered 23.12.2022 at German Clinical Trails Registre. Registration ID: DRKS00030973.

## Background

The COVID-19 pandemic has presented significant global challenges, impacting various aspects of human life. In addition to its impact on physical health, the pandemic has led to an increase in levels of depression, psychological distress, and anxiety, particularly among certain demographic groups [1, 2]. It is important to note that not all demographic groups have experienced mental health challenges to the same extent. Certain risk factors have been identified for poor mental health outcomes, including being female, belonging to a younger age group (under 40 years), experiencing unemployment or pursuing education, having chronic or psychiatric illnesses, and frequent use of social media [2].

The significance of gender as a determining factor in mental health outcomes has been acknowledged in previous studies [3–5]. Generally, women experience a higher incidence of depression and anxiety disorders throughout their lifetime. This occurrence is due to a complex interplay of biological, psychological, and socio-cultural factors [6]. These susceptibilities have been further intensified by the unique stressors of the pandemic, in addition to pre-existing gender inequalities [7]. For example, young women residing alone may have experienced intensified feelings of isolation as a consequence of the implementation of lockdown measures and social distancing guidelines, which could have resulted in elevated levels of anxiety and depression [7]. The lack of a support system in their immediate living environment can exacerbate feelings of solitude and vulnerability. Moreover, young women are frequently in the nascent stages of their careers, which may have been disrupted by the economic consequences of the pandemic, resulting in financial instability and job insecurity [8, 9]. Furthermore, a working mother who previously balanced her career with family duties might have found herself in a situation where her children were home all day due to school closures. This additional burden, coupled with the pervasive fear of the virus, financial instability, and diminished social support, significantly heightened her levels of anxiety and depression [10, 11]. These examples illustrate how the intersection of pandemic-related stressors and pre-existing gender inequalities can have a disproportionate impact on the mental health of women [12].

Given the developing challenges, it is crucial to seek interventions that support mental health and are easily accessible. Traditional mental health services, while effective, often face obstacles such as cost and limited availability [13, 14]. Therefore, solutions must involve interventions that bridge the accessibility gap and provide meaningful remedies. The increase in digitalization due to the pandemic is an advantage in this case.

This pilot study examines the potential impact of an online self-directed intervention designed to enhance mental health, with a specific emphasis on the pivotal elements of mindfulness. It is imperative that effective interventions are based on robust scientific evidence and proven methodologies, and that they are readily accessible and integrated into women's daily routines. The construct of mindfulness, defined as the awareness that emerges through paying attention on purpose, in the present moment, and non-judgementally to the unfolding of experience moment by moment [15], is at the core of this intervention. Extensive research has been conducted into the benefits of mindfulness practices for mental health. These benefits include a reduction in stress, anxiety, and depressive symptoms, as well as an improvement in overall well-being [16, 17].

Mindfulness is not merely a standalone practice; rather, it is an integral component that can enhance the efficacy of other psychological interventions. Two specific methods that have demonstrated efficacy are compassion training and gratitude training, which are based on the principles of mindfulness. Compassion training is a method that focuses on developing empathy and understanding towards oneself and others. This training has been associated with increased emotional resilience and reduced psychological distress. Gratitude training is another method that involves cultivating a sense of thankfulness and appreciation. This has been linked to greater overall well-being and reduced symptoms of depression and anxiety. Both of these approaches have shown potential in enhancing psychological well-being and alleviating symptoms of stress, anxiety, and depression [18–22].

Mindfulness is a fundamental element in the training of both compassion and gratitude. It enhances compassion training by enabling individuals to observe their thoughts and emotions without judgement, thereby fostering a kinder and more compassionate attitude towards themselves and others. Similarly, mindfulness enhances gratitude training by enabling individuals to become more aware of and appreciate the positive aspects of their lives. It can be seen, therefore, that mindfulness plays a vital role in enabling the integration and mutual reinforcement of compassion and gratitude practices [15].

The combination of compassion and gratitude training has yielded encouraging results, including notable enhancements in self-compassion [22]. Nevertheless, in order to guarantee accessibility, it is essential to ascertain whether substantial enhancements in both compassion and gratitude can be attained within a four-week timeframe through a self-directed online training programme. Furthermore, it is essential to ascertain whether these two qualities have a mutually reinforcing effect when practised in conjunction. Confirmation of this interaction is a prerequisite for future studies in this area, which could pave the way for the development of more accessible and effective interventions to enhance women's mental health through compassion and gratitude training programmes.

In conclusion, the objective of this pilot study is to examine the feasibility and efficacy of a brief, self-directed online intervention that integrates mindfulness, compassion, and gratitude training to enhance mental health outcomes in women. In doing so, it aims to contribute to the growing body of evidence supporting the use of holistic and accessible mental health interventions.

### Compassion training

Compassion training has its roots in various cultural, spiritual, and philosophical traditions. These traditions have contributed to the development of techniques aimed at promoting empathy and compassion towards oneself and others [23]. Modern compassion training is based on the integration of mindfulness-based practices and cognitive psychology, which were established in the late twentieth century [14]. This text discusses the concept of Compassion Focused Therapy (CFT) [24], which was heavily influenced by the work of psychologists such as Albert Ellis [25], Aaron Beck [26, 27], and Paul Gilbert [28]. The aim of CFT is to cultivate self-compassion and a sense of common humanity [29]. In the early twenty-first century, researchers began defining the notion of self-compassion and creating scales to quantify it. Neff's research and contributions have been significant in establishing compassion training as a legitimate element of therapy [30]. According to Neff [31, 32] and Neff and Prommier [33], cultivating self-compassion has significant implications for psychological health and overall well-being. Neuroscientific studies have supported their work, suggesting that training in compassion can result in changes to brain structure and function that relate to empathy and prosocial behaviour [34]. Neff defines self-compassion as consisting of three elements: showing kindness and understanding towards oneself instead of being harsh, self-critical, and judgmental; viewing one's experiences as part of common human experiences rather than isolating and separating; and being mindful of one's distressing thoughts and emotions, instead of excessively identifying with them [27, 35].

### Gratitude training

Gratitude is a core concept in positive psychology [36, 37] that emphasizes strengths and virtues rather than weaknesses or deficits. Hudecek et al. defines gratitude as a multifaceted concept that includes emotional, conceptual, attitudinal, and behavioral components [38]. Research in positive psychology has shown that practicing gratitude can improve well-being, promote prosocial behaviour, and alleviate mental and physical pain [39–41]. Moreover, it may serve as a protective measure against the mental health effects of the COVID-19 pandemic [42]. Focusing on and acknowledging the positive aspects of one's life can lead to increased contentment due to the experience of positive emotions [43]. This can be explained by gratitude countering the human tendency to focus on threats and negative events, which is known as the negativity bias [44]. As a result, a more balanced perspective can be achieved, leading to reduced feelings of stress and anxiety. Acknowledging the positive aspects of life can promote resilience and self-compassion [35, 45]. According to a 2021 meta-study, gratitude interventions had modest effects on symptoms of depression and anxiety; however, the findings are not consistently reported [46]. Therefore, it is worthwhile to explore interventions with more compelling evidence of efficacy. However, it is important to note that gratitude is often associated with increased subjective well-being and enhanced life satisfaction [47]. Additionally, gratitude has been linked to reduced stress levels [48] and a lower risk of anxiety and depression [49]. In summary, research has shown that both compassion training and gratitude techniques can improve well-being and reduce psychological distress. The combination of these techniques is of significant interest, particularly when delivered through online self-training due to its low cost and high level of accessibility.

### Hypotheses

The results of research conducted thus far indicate that the acquisition of predefined mindfulness skills can be significantly enhanced within a four-week period of online training. This training has been demonstrated to not only improve mindfulness but also to have a positive impact on subjective stress perceptions, which in turn has been shown to result in a reduction of stress-related symptoms and an improvement in overall well-being [50]. In light of these promising outcomes, it seems worthwhile to explore whether similar benefits can be observed when applying structured training to other positive psychological constructs, such as compassion and gratitude. Therefore, the following hypotheses are proposed:

#### Hypothesis 1

The first hypothesis is that the experimental group (EG) will exhibit significant enhancements in predetermined compassion constructs, including self-kindness, self-judgment, common humanity, isolation, mindfulness, and over-identification, after completing a four-week self-directed online course on compassion and gratitude, compared to the wait-list control group (CG).

#### Hypothesis 2

The second hypothesis is that the EG will report significant improvements in pre-defined gratitude constructs, including feelings of gratitude, attitudes towards appropriateness, behavioral shortcomings, noticing benefits/rituals, expressing gratitude, and overall attitude of gratitude, after completing a four-week self-directed online compassion and gratitude training course compared to the CG.

## Methods

### Trial design

The sample size was calculated using G-Power software, taking into account the significance level, test strength, and effect size. A total sample size of 36 persons was required, comprising 18 participants in the experimental EG and 18 in the CG, with a significance level of 0.05, a medium effect (d = 0.25), and an optimal power of 0.95. A total of 65 female participants were recruited from Instagram and using an online radomization tool randomly assigned to either an the EG or CG by the research team. The social media platform was chosen to ensure that all participants were women who met the risk factors: female, under 40, and frequent users of social media. The study targeted women under 40 who are seen as being digital natives [51], who possess the necessary skills to complete an online training program. Eligibility criteria required participants to be female, aged 18–39 at baseline, and willing to spend approximately 20 min per day practicing gratitude and compassion online. All participants in this study were German citizens. The participants were informed about their group allocation after having filled out the baseline questionniare (T1). T2 was conducted immediately after the course to measure the direct effect of the online program.

### Intervention format

A Mindfulness-Based Compassion Training (MBCT) typically consists of weekly sessions lasting 2.5–3.5 h, delivered over eight weeks. The training can be conducted remotely through Virtual Instructor Led Training (VILT) or in-person. However, the eight-week program can be time-consuming and costly. Therefore, there is a need to develop training programs that are low-threshold yet highly beneficial. Spijkerman et al. [52] conducted a meta-analysis and found that online mindfulness training significantly improves mindfulness skills, reduces stress, and enhances well-being. Additionally, previous studies have shown that predetermined mindfulness skills can be improved in as little as four weeks through online training, resulting in positive effects on perceived stress levels [50]. The significance of these findings lies in their potential to improve accessibility and ensure practicality and adaptability. It is important to assess the suitability of these findings for integrating compassion training with gratitude training.

The study analyzed a four-week self-directed online training program (called ComGrat) consisting of twenty-eight units and an introductory session. Participants received daily emails at 6:00 am, containing a brief psychoeducational text on compassion and/or gratitude, as well as a loving-kindness meditation. The participants were encouraged to read a psychoeducational text that explains the concept and research of mindfulness, loving-kindness meditation, compassion, and gratitude. They were also instructed to practice the loving-kindness meditation, which lasted about 10–12 min, at home. The first fourteen days included various meditation techniques, and for the past fortnight, participants were able to select a meditation from the previous 14 days. Participants received an email prompt at 9 pm each night to make an entry in their gratitude journal. The journal was filled out privately. Guidelines were provided at the beginning of the course on how and where to conduct the daily meditation, how to fill out the gratitude journal, and how to maintain consistency throughout the course.

### Intervention content

Compassion involves promoting empathy, kindness, and understanding towards oneself and others [53]. In the program, these skills were trained through mindfulness techniques, such as cultivating non-judgmental awareness of the present moment, developing self-kindness through self-compassion, recognizing one's shared humanity, applying mindfulness during challenging situations, and participating in empathy-enhancing exercises to connect emotionally with others. Additionally, compassion training frequently includes exercises such as expressing gratitude, forgiveness, and performing random acts of kindness, which were also incorporated into the program. To foster compassion, the program utilized loving-kindness meditation, as well as regular self-reflection through meditation or journaling [54–56].

#### Loving kindness meditation

Loving kindness meditation is also known as metta meditation [57]. It is a contemplative practice in which the practitioner engages in a systematic and deliberate process of generating feelings of compassion and benevolence for themselves, loved ones, acquaintances, strangers and people with whom they may be in conflict. It originated in the Buddhist tradition but has since been adapted to various settings with the aim of promoting compassion.

Comprehensive research has provided compelling evidence for the positive influence of practicing meditation, specifically on personal compassion skills [58]. However, a meta-analysis conducted by Gu et al. [59] suggests that the impact of general compassion training remains uncertain, with few such training programs demonstrating an effect. However, the meta-analysis shows that the practice of loving-kindness meditation has a significant positive impact on life satisfaction. Research has demonstrated that this type of meditation can lead to improvements in resilience, contentment, personal proficiency, and reduction in depression, anxiety, and stress related to stress. Loving-kindness meditation is considered crucial for cultivating a compassionate mindset [60–62]. In conclusion, loving-kindness meditation plays an important role in fostering a kind and caring approach. The meditation was practiced daily in different variations throughout the program and was accessed online via a hyperlink.

#### Journaling

Journaling is a therapeutic technique commonly used in various therapeutic contexts. It involves writing down personal thoughts, feelings, and experiences to facilitate self-reflection, emotional processing, and personal development. Several studies have suggested positive psychological effects on self-efficacy and locus of control [63]. In the early 2000 s, gratitude journaling emerged as a prominent phenomenon within the domain of positive psychology, as evidenced by the growing interest and research conducted by Seligman and his colleagues [64]. A gratitude journal is a tool whereby individuals regularly jot down things for which they are thankful. It aims to redirect focus away from deficiencies and towards acknowledging and valuing existing blessings, fostering a more optimistic perspective. Research has indicated that maintaining a gratitude journal may generate greater humility [65] and enhance overall welfare [66]. The participants were instructed to maintain a journal, either digital or analog, and use it to reflect on gratitude daily in the evening.

### Outcome measures

Participants completed pre- and post-intervention questionnaires at T1 and T2, respectively. These questionnaires assessed their levels of self-compassion and gratitude using the German Self-Compassion Scale (SCS-G) [67, 68] and the German Multi-Component Gratitude Measure (MCGM) [49, 69], respectively. The SCS consists of 26 items with a five-point Likert response format ranging from'almost never'(1) to'almost always'(5). This scale assesses six key aspects of self-compassion: self-kindness (e.g., I’m kind to myself when I’m experiencing suffering), common humanity (e.g., I try to see my failings as part of the human condition) self-judgment (e.g., I’m disapproving and judgmental about my own flaws and in-adequacies), isolation (e.g., When I do not succeed at something that is important to me, I often feel isolated in my failure), mindfulness (e.g. I practice mindfulness by attempting to maintain a balanced perspective when faced with painful situations) and over-identification I avoid over-identifying with my failures by not allowing feelings of inadequacy to consume me). For the accurate interpretation of results, it is imperative to reverse the scores for the Isolation, Self-Judgment, and Over-Identification subscales, as higher scores on these subscales initially indicate lower self-compassion [70]. The SCS provides both a total score and individual scores for each subscale. The total self-compassion score is calculated by averaging the scores across all items on the scale. Higher scores across all subscales, following the requisite reverse scoring, reflect superior self-compassion, thus providing a comprehensive assessment of an individual's self-compassionate tendencies. Self-compassion entails treating oneself with kindness in the face of pain or failure, as opposed to being harshly self-critical. It also involves recognising that suffering and personal inadequacy are part of the shared human experience and thus treating oneself with understanding and forgiveness. A high score on the total self-compassion scale or subscales is indicative of a higher level of self-compassion, which is associated with optimal emotional functioning and resilience. Conversely, a low score may indicate a necessity for interventions designed to enhance self-compassion, such as mindfulness-based therapies or self-compassion training programmes. The SCS demonstrates high internal consistency with a rating of 0.92. Furthermore, studies [67, 68] indicate that the scale has sound construct validity and test–retest reliability.

The MCGM is a comprehensive method for measuring gratitude that includes both emotional and behavioural aspects of gratitude, developed by Morgan et al. [69]. The German version of the Multi-Component Gratitude Measure (MCGM), developed by Hudecek, Blabst, Morgen, and Lermer in 2021 [49], is a comprehensive tool for assessing gratitude. The scale comprises 26 items that assess five distinct dimensions. The five dimensions of the German version of the Multi-Component Gratitude Measure (MCGM) are as follows: Attitudes of Adequacy (ATA), Attitudes of Gratitude (AOG), Mindfulness (BS), Expressing Gratitude (EOG), and Recognition of Rituals/Benefits (RNB). Each item is rated on a Likert scale from 1 (strongly disagree) to 7 (strongly agree), with each subscale being scored separately. It is confirmed that all items within a subscale are positively keyed, indicating that a higher score on each item is indicative of a higher level of gratitude. In the event that any items are negatively keyed (where a higher score would indicate a lower level of gratitude), the score of these items is reversed [69]. Higher scores indicate a greater presence of the specific aspect of gratitude, such as the frequency of expressions of gratitude or a high level of mindfulness regarding gratitude [49]. Higher scores on the MCGM indicate a higher degree of gratitude across its multiple dimensions, suggesting stronger attitudes of adequacy and gratitude, greater mindfulness, more frequent expressions of gratitude, and better recognition of rituals and benefits. Conversely, lower scores indicate a lesser degree of these aspects of gratitude. The results of studies utilising the MCGM indicate that individuals with higher scores in these dimensions tend to report greater overall well-being and positive psychological outcomes, including improved mental health, resilience, and social support. The different dimensions of the MCGM showed satisfactory to very good internal consistency (Cronbach's alpha = 0.73–0.90). The dimension with the highest value was'feelings of gratitude'(α = 0.90), while the weakest value was for the first-order attitude-related factor (α = 0.73). McDonald's omega was also in a good range for all dimensions (ω = 0.78–0.90) [64]. In addition to the questionnaires, pre-training demographics were collected, including, education, profession, number of children, experience with gratitude and compassion training, age and gender (female or otherwise) to ensure eligibility. After the training, respondents were asked about the frequency of daily meditation and gratitude diary entries.

### Statistical methods

We analyzed the collected data using JASP. We used repeated measures ANOVA to test the hypotheses to examine the differences between the EG and the CG over time [71]. We used Levene's test for equality of variance to test the assumptions for the analysis of normal distribution and homogeneity of variance, and the Shapiro–Wilk test for normal distribution. If the assumption of homogeneity of variance was confirmed, post-hoc multiple comparisons were performed using Tukey's method. If homogeneity was violated, we performed post-hoc tests using Holm's method. We only calculated planned comparisons based on our hypothesis. According to our a priori power analysis, we set a significance level of 0.05 for repeated measures ANOVA and its corresponding post hoc tests. This ensures that the power of all analyses is at least 0.85 or higher. Partial eta-squared was reported to assess effect sizes. The following statistics were reported to assess the strength of the significant mean differences observed in the Tukey HSD post-hoc comparisons: The mean differences and their 95% CIs for the specific comparison, and Cohen's d as an established measure of effect size for group comparisons. The Bonferroni correction was used to adjust the level of the CIs to reduce the overall probability of false positives when testing multiple hypotheses simultaneously. Cohen has provided benchmarks for defining small (η2 = 0.01), medium (η2 = 0.06) and large (η2 = 0.14) effects for eta-squared, and d is suggested to be interpreted as small ≥ 0.1, medium ≥ 0.3 or large ≥ 0.5. Pearson's correlation coefficient (*r*) was used for normative correlations, whereas Spearman's Rho was used for non-normative data. The effect size for both Pearson (*r*) and Spearman (ρ) was interpreted in a similar way. An effect size of less than 0.01 indicated no effect, while an effect size of 0.1 indicated a small effect. A medium effect was indicated by an effect size of 0.3, and an effect size of 0.5 indicated a large effect (jasp-stats.org, 2023).

## Results

The randomized controlled trial involved 51 female participants aged between 22 and 39 years old, all of whom provided informed written consent. Recruitment took place from February to April 2023. The study consisted of two assessment periods, baseline (T1) and post-intervention (T2), with measurements taken in April and May 2023, respectively. Random assignment to the EG and CG resulted in similar age distribution subsamples. To evaluate equity in socio-demographic variables between the two groups, independent t-tests were conducted for age and educational level. The results showed no significant differences, indicating sample equivalence for hypothesis testing. The study involved 26 female participants (*n* = 26) in the EGwith a mean age of 29.4 years (SD = 3.88), and 25 female participants (*n* = 25) in the CG with a mean age of 30.4 years (SD = 4.05) (refer to Table 1). No notable discrepancies were identified with regard to the participants'experiences with gratitude and compassion training, educational attainment, the number of children they had, their material circumstances or profession.

**Table 1:** Baseline demographic of age

Descriptive statistics		
	How old are you?	
	EG	CG
Valid	26	25
Missing	0	0
Mean	29.36	30.36
Std. Deviation	3.88	4.05
Minimum	21.00	23.00
Maximum	37.00	39.00

All 51 participants completed the questionnaires at both T1 and T2 due to regular reminder emails after the intervention, and there were no dropouts.

### Perquisitions

Normal distribution could be assumed for both main scales and most sub-scales (*p* > 0.05), except for SCS Self-Kindness, MCGM-FOG, MCGM-EOG, and MCGM-AOG (Table). However, ANOVA is relatively robust in regard to normal distribution violation [61] Variance homogeneity could be assumed for all main scales and sub-scales, except for MCGM-AOG. In the case of the latter, the post hoc test was conducted in accordance with Holm. For all others, the post hoc test was conducted in accordance with Tukey.

### Results of test for replication—self compassion

The study analyzed the changes over time in the main Self-Compassion Scale (SCS) and its six sub-scales, namely self-kindness, self-judgment, common humanity, isolation, mindfulness, and over-identification, between the EG and CG.

#### SCS—Complete

The analysis revealed that there was a normal distribution and variance homogeneity. A repeated measures ANOVA was conducted, which showed a statistically significant difference between mean performance levels across measurements, F(1,49) = 26.449, *p* < 0.001, partial η^2^ = 0.351. Additionally, there was a significant interaction effect between time and group on the primary SCS score scale, F(1,49) = 59.135, *p* < 0.001, η^2^p = 0.547. Post hoc tests confirmed significant implicit differences between the EG and the CG (t(51) = 4.527, *p* = 0.001, d = 1.268). The intervention resulted in a significant improvement of scores from T1 to T2 in the EG (t(26) = −9.164, *p* < 0.001), with a mean difference (MD) of −0.883 points and a 95% CI of [−1.148, −0.618], d = −1.288(refer to Table 2).

**Table 2:** Post Hoc Tests SCS Complete

Post Hoc Comparisons—Group ✻ Time													
			**95% CI for Mean Difference**					**95% CI for Cohen's d**					
		**Mean Difference**	**Lower**	**Upper**	**SE**	**t**	**Cohen's d**	**Lower**	**Upper**	**p**_**tukey**_		**p**_**bonf**_	
EG, T1	CG, T1	−0.189	−0.712	0.334	0.192	−0.984	−0.276	−1.042	0.491	0.759		1.000	
	EG, T2	−0.883	−1.148	−0.618	0.096	−9.164	−1.288	−1.810	−0.766	<.001	***	<.001	***
	CG, T2	−0.014	−0.537	0.509	0.192	−0.071	−0.020	−0.783	0.743	1.000		1.000	
CG, T1	EG, T2	−0.694	−1.217	−0.171	0.192	−3.614	−1.012	−1.825	−0.200	0.003	**	0.004	**
	CG, T2	0.175	−0.095	0.445	0.098	1.784	0.256	−0.141	0.652	0.293		0.484	
EG, T2	CG, T2	0.869	0.346	1.392	0.192	4.527	1.268	0.429	2.107	<.001	***	<.001	***

*P*-value and confidence intervals adjusted for comparing a family of 6 estimates (confidence intervals corrected using the bonferroni method)

^**^p <.01

^***^p <.001

**Fig. 1:**
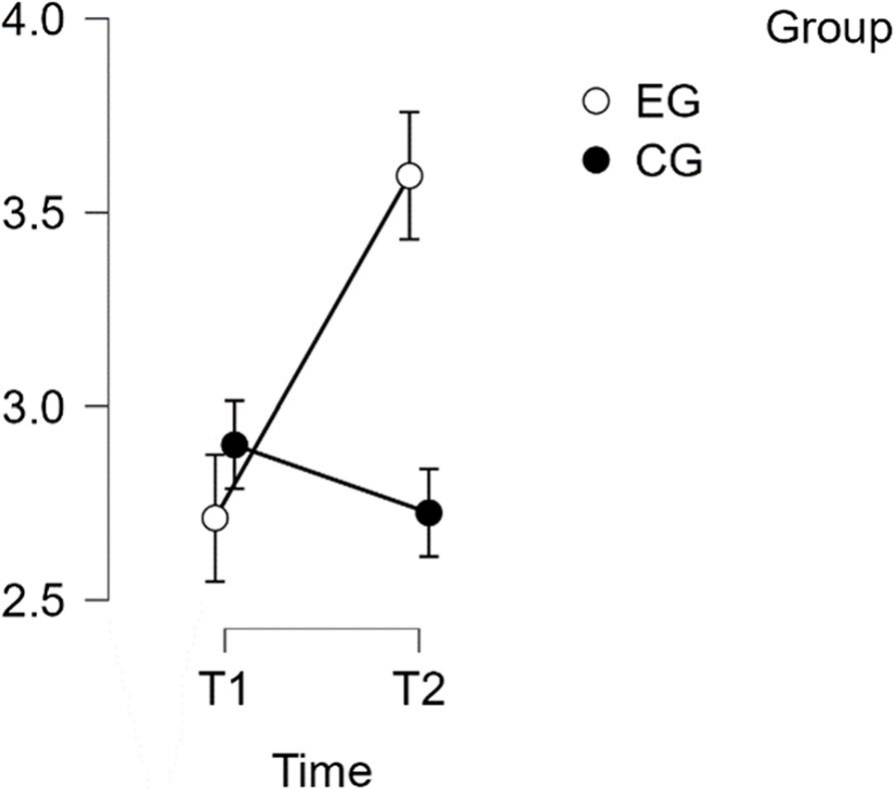
Estimated marginal means for Self-Compassion Subscale complete score

This effect size is considered large. Therefore, H1 is confirmed (refer to Fig. 1).

#### SCS self kindness subscale

The analysis showed a violation of normal distribution and homogeneity of variance. The repeated measures ANOVA revealed a statistically significant difference in mean performance levels between measurements (F(1,49) = 23.185, *p* < 0.001, partial η^2^ = 0.321). The statistical significance of the interaction between time and group on the sub-scale Self-Kindness of the SCS score was confirmed (F(1,49) = 49.113, *p* < 0.001, η^2^p = 0.501). Post-hoc tests revealed significant implicit differences between the EG and CG groups (t(51) = 4.134, *p* = 0.001, d = 1.158). The intervention resulted in a noteworthy increase in scores from T1 to T2 in the EG. The difference was statistically significant, with t(26) = −8.443, *p* < 0.001 and a mean difference (MD) of −1.208 points, 95% CI [−1.601, −0.814], d = −1.277, indicating a large effect size (refer to Table 3).

**Table 3:** Post hoc test SCS—self kindness subscale

Post Hoc Comparisons—Group ✻ Time										
**Mean Difference**			**95% CI for Mean Difference**					**95% CI for Cohen's d**		
			**Lower**	**Upper**	**SE**	**t**	**Cohen's d**	**Lower**	**Upper**	**p**_**tukey**_
EG, T1	CG, T1	−0.336	−1.057	0.384	0.265	−1.269	−0.356	−1.124	0.413	0.585
	EG, T2	−1.208	−1.601	−0.814	0.143	−8.443	−1.277	−1.818	−0.736	<.001
	CG, T2	−0.112	−0.833	0.608	0.265	−0.424	−0.119	−0.882	0.644	0.974
CG, T1	EG, T2	−0.871	−1.592	−0.151	0.265	−3.289	−0.921	−1.724	−0.118	0.009
	CG, T2	0.224	−0.177	0.625	0.146	1.536	0.237	−0.188	0.661	0.425
EG, T2	CG, T2	1.095	0.375	1.816	0.265	4.134	1.158	0.332	1.984	<.001

*P*-value and confidence intervals adjusted for comparing a family of 6 estimates (confidence intervals corrected using the bonferroni method)

**Fig. 2:**
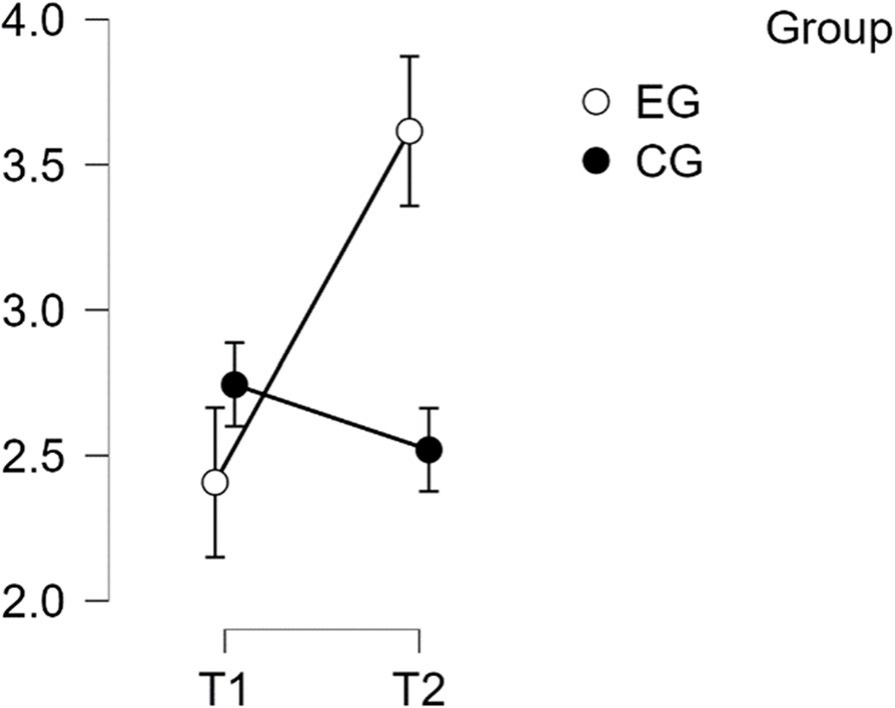
Estimated marginal means for self-compassion subscale self kindness

These findings confirm H1 regarding Self Kindness (refer to Fig. 2).

#### SCS—self judgement subscale

The analysis showed normal distribution and homogeneity of variance. The repeated measures ANOVA indicated a statistically significant difference in mean performance levels between measurements, F(1,49) = 17.373, *p* < 0.001, partial η^2^ = 0.262. The statistical significance of the time * group interaction on the Self-Judgement sub-scale of the SCS score was also confirmed, F(1,49) = 27.113, *p* < 0.001, η^2^p = 0.501. Post-hoc tests confirmed implicit differences between EG and CG (t(51) = 4.134, *p* = 0.001, d = 1.158). The intervention resulted in a significant increase in scores from T1 to T2 in EG (t(26) = −6.695, *p* < 0.001) with a mean difference (MD) of −0.938 points, 95% CI [−1.324, −0.553], d = −1.081, which is considered a large effect (See Table 4).

**Table 4:** Post hoc test SCS—self judgement subscale

Post Hoc Comparisons—Group ✻ RM Factor 1										
			**95% CI for Mean Difference**					**95% CI for Cohen's d**		
		**Mean Difference**	**Lower**	**Upper**	**SE**	**t**	**Cohen's d**	**Lower**	**Upper**	**p**_**tukey**_
EG, T1	CG, T1	−0.014	−0.674	0.647	0.243	−0.056	−0.016	−0.777	0.746	1.000
	EG, T2	−0.938	−1.324	−0.553	0.140	−6.695	−1.081	−1.611	−0.551	<.001
	CG, T2	0.090	−0.570	0.751	0.243	0.372	0.104	−0.658	0.866	0.982
CG, T1	EG, T2	−0.925	−1.586	−0.264	0.243	−3.803	−1.065	−1.881	−0.250	0.002
	CG, T2	0.104	−0.289	0.497	0.143	0.728	0.120	−0.329	0.568	0.886
EG, T2	CG, T2	1.029	0.368	1.690	0.243	4.231	1.185	0.357	2.013	<.001

*P*-value and confidence intervals adjusted for comparing a family of 6 estimates (confidence intervals corrected using the bonferroni method)

**Fig. 3:**
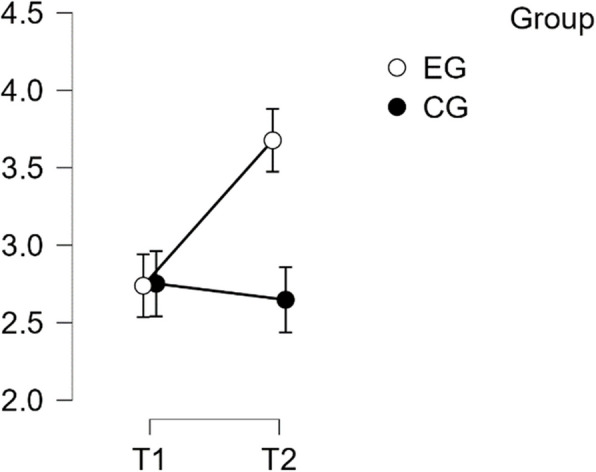
Estimated marginal means for self-compassion subscale self-judgement

Therefore, H1 was confirmed in regards to Self Judgement (see Fig. 3).

#### SCS—common humanity subscale

The analysis showed normal distribution and homogeneity of variance. The repeated measures ANOVA indicated no statistically significant difference in mean performance levels between measurements, F(1,49) = 3.733, *p* = < 0.059, partial η^2^ = 0.071. However, the time * group interaction on the sub-scale Common Humanity of the SCS score was found to be statistically significant, F(1,49) = 32.856, *p* = < 0.001, η^2^p = 0.401. Post-hoc tests showed significant implicit differences between the EG and the CG (t(51) = 5.218, *p* = 0.001, d = 1.462). The intervention resulted in a significant increase in scores from T1 to T2 in the EG (t(26) = −5.473, *p* < 0.001), with a mean difference (MD) of −0.827 points, 95% CI [−1.242, −0.411], d = −1.072, indicating a large effect (refer to Table 5).

**Table 5:** Post hoc test SCS—common humanity subscale

Post Hoc Comparisons—Group ✻ Time										
			**95% CI for Mean Difference**					**95% CI for Cohen's d**		
		**Mean Difference**	**Lower**	**Upper**	**SE**	**t**	**Cohen's d**	**Lower**	**Upper**	**p**_**tukey**_
EG, T1	CG, T1	−0.109	−0.694	0.476	0.216	−0.505	−0.142	−0.901	0.618	0.958
	EG, T2	−0.827	−1.242	−0.411	0.151	−5.473	−1.072	−1.678	−0.466	<.001
	CG, T2	0.301	−0.284	0.886	0.216	1.392	0.390	−0.376	1.156	0.508
CG, T1	EG, T2	−0.718	−1.303	−0.133	0.216	−3.321	−0.930	−1.730	−0.130	0.007
	CG, T2	0.410	−0.014	0.834	0.154	2.661	0.531	−0.028	1.091	0.050
EG, T2	CG, T2	1.128	0.543	1.713	0.216	5.218	1.462	0.604	2.319	<.001

*P*-value and confidence intervals adjusted for comparing a family of 6 estimates (confidence intervals corrected using the bonferroni method)

**Fig. 4:**
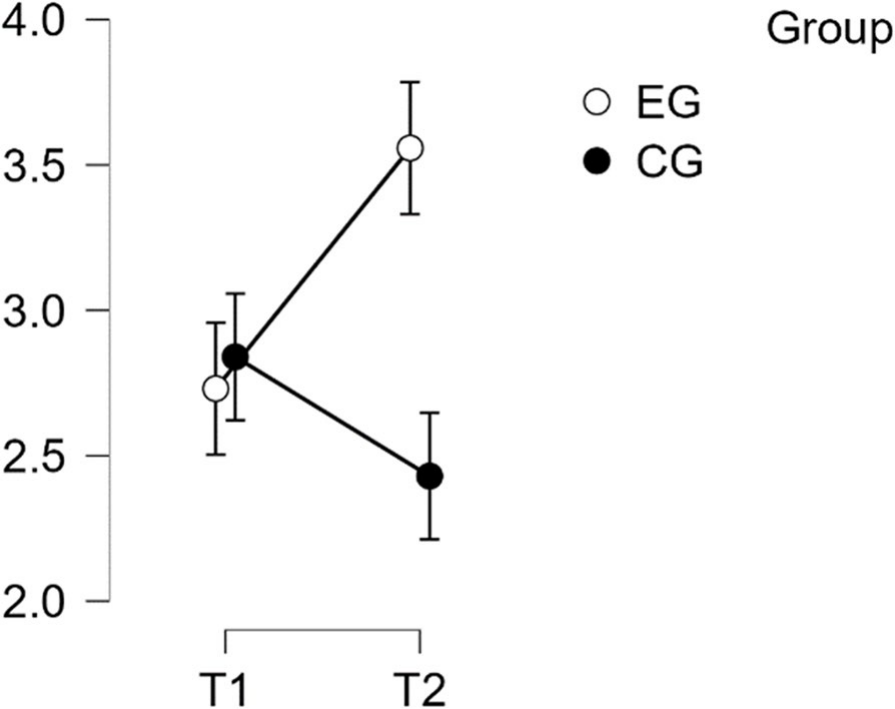
Estimated marginal means for self-compassion subscale common humanity

Therefore, H1 was confirmed regarding Common Humanity (see Fig. 4).

#### SCS – isolation subscale

The analysis showed that the distribution was normal, and the variance was homogeneous. The repeated measures ANOVA revealed a statistically significant difference in mean performance levels between measurements (F(1,49) = 9.043, *p* < 0.004, partial η^2^ = 0.154). The statistical significance of the interaction between time and group on the sub-scale Isolation of the SCS score was also determined (F(1,49) = 14.992, *p* < 0.001, η^2^p = 0.234). Post-hoc analysis confirmed significant differences between the EG and the CG (t(51) = 2.859, *p* = 0.028, d = 0.801). The intervention resulted in a significant improvement in scores from T1 to T2 within the EG (t(26) = −4.913, *p* < 0.001). The mean difference (MD) was −0.875 points, 95% CI [−1.365, −0.385], d = −0.870, indicating a large effect (refer to Table 6).

**Table 6:** Post hoc test SCS – isolation subscale

Post Hoc Comparisons—Group ✻ RM Factor 1										
			**95% CI for Mean Difference**					**95% CI for Cohen's d**		
		**Mean Difference**	**Lower**	**Upper**	**SE**	**t**	**Cohen's d**	**Lower**	**Upper**	**p**_**tukey**_
EG, T1	CG, T1	−0.180	−0.944	0.584	0.282	−0.639	−0.179	−0.941	0.582	0.919
	EG, T2	−0.875	−1.365	−0.385	0.178	−4.913	−0.870	−1.407	−0.334	<.001
	CG, T2	−0.070	−0.834	0.694	0.282	−0.249	−0.070	−0.830	0.691	0.995
CG, T1	EG, T2	−0.695	−1.459	0.069	0.282	−2.468	−0.691	−1.475	0.092	0.074
	CG, T2	0.110	−0.389	0.609	0.182	0.606	0.109	−0.382	0.600	0.930
EG, T2	CG, T2	0.805	0.041	1.569	0.282	2.859	0.801	0.010	1.592	0.028

*P*-value and confidence intervals adjusted for comparing a family of 6 estimates (confidence intervals corrected using the bonferroni method)

**Fig. 5:**
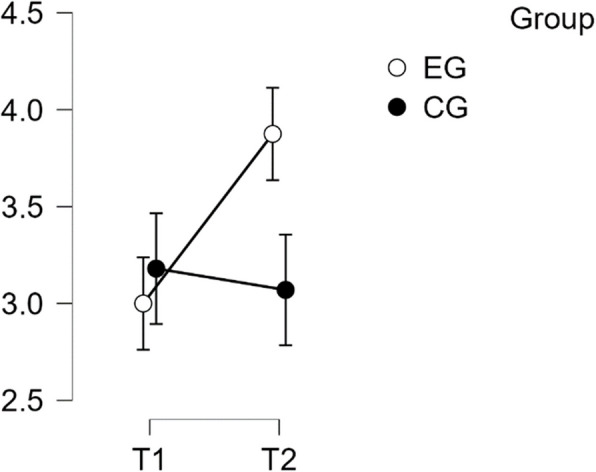
Estimated marginal means for self-compassion subscale isolation

Therefore, H1 was confirmed in regards to Isolation (see Fig. 5).

#### SCS – mindfulness subscale

The statistical analysis showed that the distribution was normal and the variance was homogeneous. The repeated measures ANOVA showed a statistically significant difference in mean performance levels between measurements (F(1,49) = 16.791, *p* = < 0.001, partial η^2^ = 0.255). An interaction between time and group was observed for the'Mindfulness'subscale of the SCS score (F(1,49) = 23.769, *p* < 0.001, η^2^p = 0.327). Post-hoc analysis revealed significant differences between the EG and the CG (t(51) = 3.148, *p* = 0.013, d = 0.882). The intervention resulted in a significant increase in T2 scores in the EG, t(26) = −6.408, *p* < 0.001, MD of −0.923 points, 95% CI [−1.319, −0.527], d = −1.100, representing a large effect size (refer to Table 7).

**Table 7:** Post hoc test SCS – mindfulness subscale

Post Hoc Comparisons—Group ✻ RM Factor 1										
			**95% CI for Mean Difference**					**95% CI for Cohen's d**		
		**Mean Difference**	**Lower**	**Upper**	**SE**	**t**	**Cohen's d**	**Lower**	**Upper**	**p**_**tukey**_
EG, T1	CG, T1	−0.263	−0.901	0.375	0.235	−1.119	−0.313	−1.079	0.452	0.679
	EG, T2	−0.923	−1.319	−0.527	0.144	−6.408	−1.100	−1.655	−0.545	<.001
	CG, T2	−0.183	−0.821	0.455	0.235	−0.779	−0.218	−0.981	0.545	0.864
CG, T1	EG, T2	−0.660	−1.298	−0.022	0.235	−2.808	−0.786	−1.577	0.004	0.032
	CG, T2	0.080	−0.324	0.484	0.147	0.545	0.095	−0.380	0.571	0.948
EG, T2	CG, T2	0.740	0.102	1.378	0.235	3.148	0.882	0.084	1.680	0.013

*P*-value and confidence intervals adjusted for comparing a family of 6 estimates (confidence intervals corrected using the bonferroni method)

**Fig. 6:**
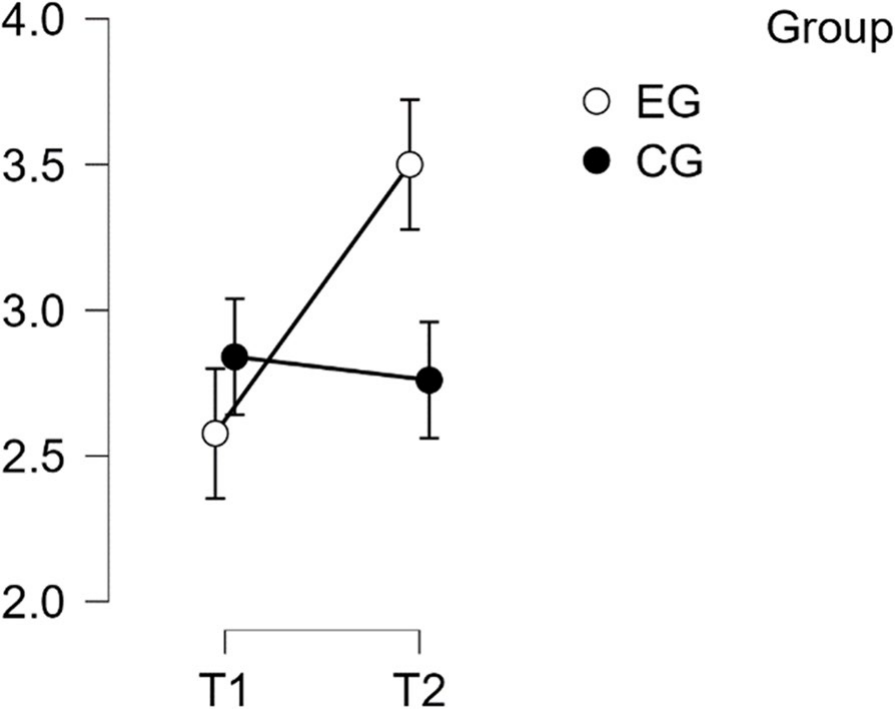
Estimated marginal means for self-compassion subscale mindfulness

Hypothesis H1 was confirmed concerning Mindfulness (see Fig. 6).

#### SCS—over identification subscale

The analysis showed a normal distribution and even variance. The repeated measures ANOVA revealed a statistically significant difference in mean performance levels between measurements (F(1,49) = 5.732, *p* = 0.021, partial η^2^ = 0.105). The statistical significance of the interaction between time and group on the Over-Identification subscale of the SCS score was also established (F(1,49) = 15.948, *p* < 0.001, η^2^p = 0.246). However, the post-hoc tests did not confirm any inherent differences between the EG and the CG (t(51) = 2.222, *p* = 0.127). Nevertheless, the intervention resulted in a substantial improvement in scores from T1 to T2 in the EG. The statistical analysis showed a significant increase, t(26) = −4.562, *p* < 0.001, with a mean difference (MD) of −0.519 points, 95% CI [−0.832, −0.206], d = −0.776, indicating a large effect size (see Table 8).

**Table 8:** Post Hoc Test SCS—Over-Identification Subscale

Post Hoc Comparisons—Group ✻ RM Factor 1										
			**95% CI for Mean Difference**					**95% CI for Cohen's d**		
		**Mean Difference**	**Lower**	**Upper**	**SE**	**t**	**Cohen's d**	**Lower**	**Upper**	**p**_**tukey**_
EG, T1	CG, T1	−0.233	−0.742	0.276	0.187	−1.241	−0.348	−1.114	0.419	0.603
	EG, T2	−0.519	−0.832	−0.206	0.114	−4.562	−0.776	−1.284	−0.267	<.001
	CG, T2	−0.103	−0.612	0.406	0.187	−0.548	−0.153	−0.915	0.608	0.947
CG, T1	EG, T2	−0.287	−0.795	0.222	0.187	−1.528	−0.428	−1.198	0.341	0.426
	CG, T2	0.130	−0.189	0.449	0.116	1.120	0.194	−0.280	0.668	0.679
EG, T2	CG, T2	0.417	−0.092	0.925	0.187	2.222	0.622	−0.157	1.402	0.127

*P*-value and confidence intervals adjusted for comparing a family of 6 estimates (confidence intervals corrected using the bonferroni method)

**Fig. 7:**
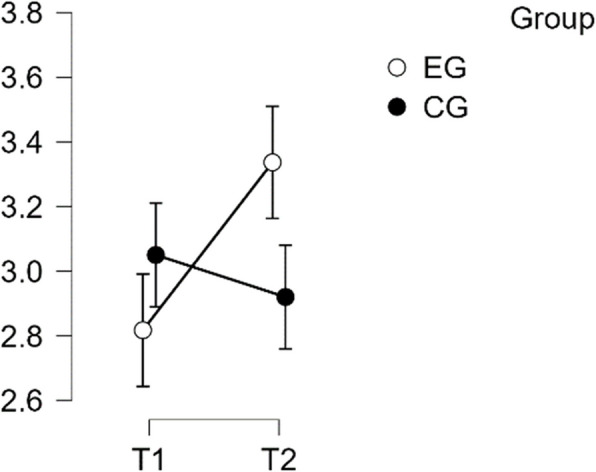
Estimated marginal means for self-compassion subscale over identification

Hypothesis H1 was only partially confirmed regarding Over-Identification (see Fig. 7).

### Results of test for replication – gratitude

The study analyzed the divergence between the EG and CG over time in relation to the primary Multi-Component Gratitude Measure (MCGM) scale and its six sub-scales: Feelings of gratitude (FOG), Attitudes to appropriateness (ATA), Behavioral shortcomings (BS), Rituals/Noticing benefits (RNB), Expression of gratitude (EOG), and Attitude of gratitude (AOG).

#### MCGM – complete

The analysis showed that the distribution was normal and the variance was homogeneous. The repeated measures ANOVA revealed a statistically significant difference in mean performance levels between measurements (F(1,49) = 21.946, *p* < 0.001, partial η^2^ = 0.309). The statistical significance of the interaction between time and group on the primary scale MCGM score was also established (F(1:49) = 46.287, *p* < 0.001, η^2^p = 0.486). Subsequent post-hoc assessments confirmed the inherent disparities between the EG and the CG (t(51) = 4.113, *p* < 0.001). The intervention resulted in a noteworthy increase in scores from T1 to T2 in the EG, t(26) = −8.204, *p* < 0.001, with a mean difference (MD) of −0.648 points, 95% CI [−0.866, −0.431], d = −1.113 (see Table 9).

**Table 9:** Post hoc test MCGM complete

Post Hoc Comparisons—Group ✻ RM Factor 1										
			**95% CI for Mean Difference**					0.934		
		**Mean Difference**	<.001	**Upper**	**SE**	**t**	**Cohen's d**	**Lower**	**Upper**	0.934
EG, T1	CG, T1	−0.097	0.999	0.348	0.163	−0.593	−0.166	−0.931	−0.931	<.001
	EG, T2	−0.648	0.007	−0.431	0.079	−8.204	−1.113	−1.593	−1.593	0.999
	CG, T2	0.023	0.455	0.468	0.163	0.140	0.039	−0.724	−0.724	0.007
CG, T1	EG, T2	−0.552	<.001	−0.107	0.163	−3.380	−0.947	−1.754	−1.754	0.455
	CG, T2	0.120	−0.102	0.341	0.081	1.484	0.205	−0.176	−0.176	<.001
EG, T2	CG, T2	0.671	0.226	1.116	0.163	4.113	1.152	0.325	0.325	<.001

*P*-value and confidence intervals adjusted for comparing a family of 6 estimates (confidence intervals corrected using the bonferroni method)

**Fig. 8:**
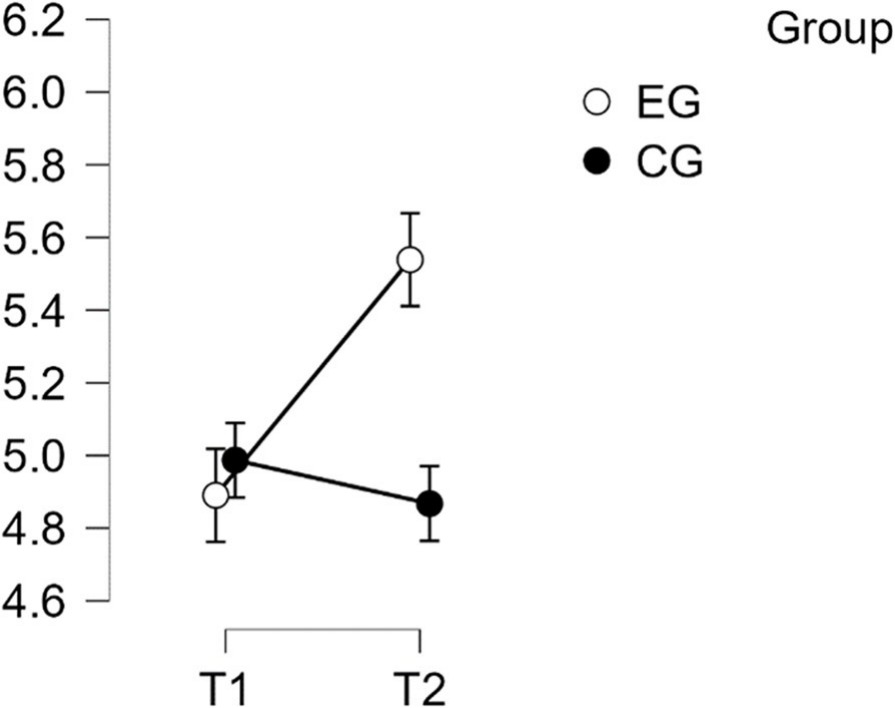
Estimated marginal means for multi-component gratitude measure scale total

This outcome is classified as a large effect, confirming Hypothesis H2 (see Fig. 8).

#### MCGM – FOG subscale: feelings of gratitude

The normality of the distribution was violated, and variance homogeneity was confirmed in the analysis. The repeated measures ANOVA revealed a statistically significant difference in mean performance levels between measurements, F(1,49) = 22.021, *p* < 0.001, partial η^2^ = 0.310. An interaction between time and group was detected in the FOG subscale of the MCGM, F(1,49) = 21.931, *p* < 0.001, η^2^p = 0.309. However, the post-hoc tests did not reveal any significant differences between the EG and the CG, t(51) = 1.998, *p* = 0.200. The intervention resulted in a noteworthy improvement in scores for the EG from T1 to T2, t(26) = −6.696, *p* < 0.001 with a mean difference (MD) of −0.781 points, 95% CI [−1.101, −0.460], d = −0.857, which is characterized as a large effect (refer to Table 10).

**Table 10:** Post hoc test MCGM – FOG subscale

Post Hoc Comparisons—Group ✻ RM Factor 1										
			**95% CI for Mean Difference**					**95% CI for Cohen's d**		
		**Mean Difference**	**Lower**	**Upper**	**SE**	**t**	**Cohen's d**	**Lower**	**Uppe**	**p**_**tukey**_
EG, T1	CG, T1	−0.270	−0.966	0.426	0.255	−1.059	−0.297	−1.065	0.472	0.715
	EG, T2	−0.781	−1.101	−0.460	0.117	−6.696	−0.857	−1.279	−0.436	<.001
	CG, T2	−0.271	−0.967	0.425	0.255	−1.062	−0.298	−1.066	0.471	0.714
CG, T1	EG, T2	−0.511	−1.207	0.186	0.255	−2.001	−0.560	−1.340	0.219	0.199
	CG, T2	−8.000 × 10^–4^	−0.328	0.326	0.119	−0.007	−8.782 × 10^–4^	−0.357	0.355	1.000
EG, T2	CG, T2	0.510	−0.186	1.206	0.255	1.998	0.560	−0.220	1.339	0.200

*P*-value and confidence intervals adjusted for comparing a family of 6 estimates (confidence intervals corrected using the bonferroni method)

**Fig. 9:**
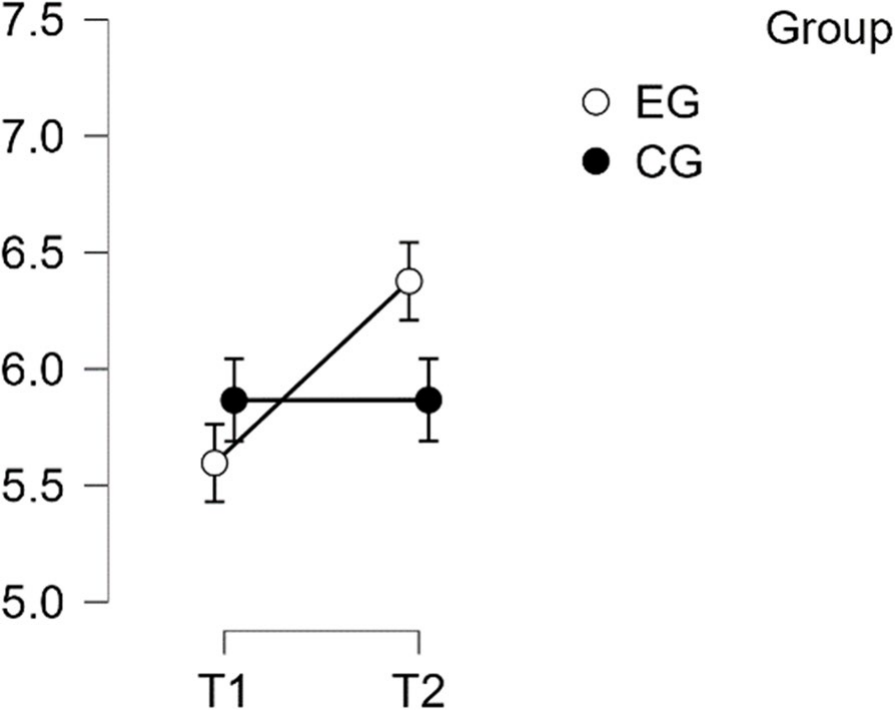
Estimated marginal means for multi-component gratitude measure subscale feelings of gratitude

Therefore, hypothesis H2 was only partially confirmed in gegards to the FOG Subscale (see Fig. 9).

#### MCGM – ATA subscale: attitudes to appropriateness

The analysis was used to determine the normality of distribution and homogeneity of variance. In relation to the MCGM sub-scale ATA, there was no statistically significant interaction between time and group (F(1:49) = 1.582, *p* = 0.214, η^2^p = 0.031). Therefore, hypothesis H2b was not confirmed.

#### MCGM – BS subscale: behavioral shortcomings

The analysis showed that there was a normal distribution and homogeneity of variance. The statistical significance of the interaction between time and group on the sub-scale BS was confirmed (F(1:49) = 26.336, *p* < 0.001, η^2^p = 0.350). Post-hoc tests confirmed significant differences between the EG and the CG (t(51) = 3.871, *p* = 0.001). The intervention resulted in a significant improvement in scores from T1 to T2 in the EG, t(26) = −7.225, *p* < 0.001, with a mean difference (MD) of −1.375, 95% CI −1.898 to −0.852, d = −1.056, indicating a large effect size (refer to Table 11).

**Table 11:** Post Hoc Test MCGM – BS Subscale

Post Hoc Comparisons—Group ✻ RM Factor 1										
			**95% CI for Mean Difference**					**95% CI for Cohen's d**		
		**Mean Difference**	**Lower**	**Upper**	**SE**	**t**	**Cohen's d**	**Lower**	**Upper**	**p**_**tukey**_
EG, T1	CG, T1	0.018	−0.976	1.011	0.365	0.048	0.014	−0.749	0.776	1.000
	EG, T2	−1.375	−1.898	−0.852	0.190	−7.225	−1.056	−1.548	−0.563	<.001
	CG, T2	0.038	−0.956	1.031	0.365	0.103	0.029	−0.734	0.792	1.000
CG, T1	EG, T2	−1.393	−2.386	−0.399	0.365	−3.817	−1.069	−1.886	−0.252	0.002
	CG, T2	0.020	−0.514	0.554	0.194	0.103	0.015	−0.390	0.421	1.000
EG, T2	CG, T2	1.413	0.419	2.406	0.365	3.871	1.084	0.266	1.903	0.001

*P*-value and confidence intervals adjusted for comparing a family of 6 estimates (confidence intervals corrected using the bonferroni method)

**Fig. 10:**
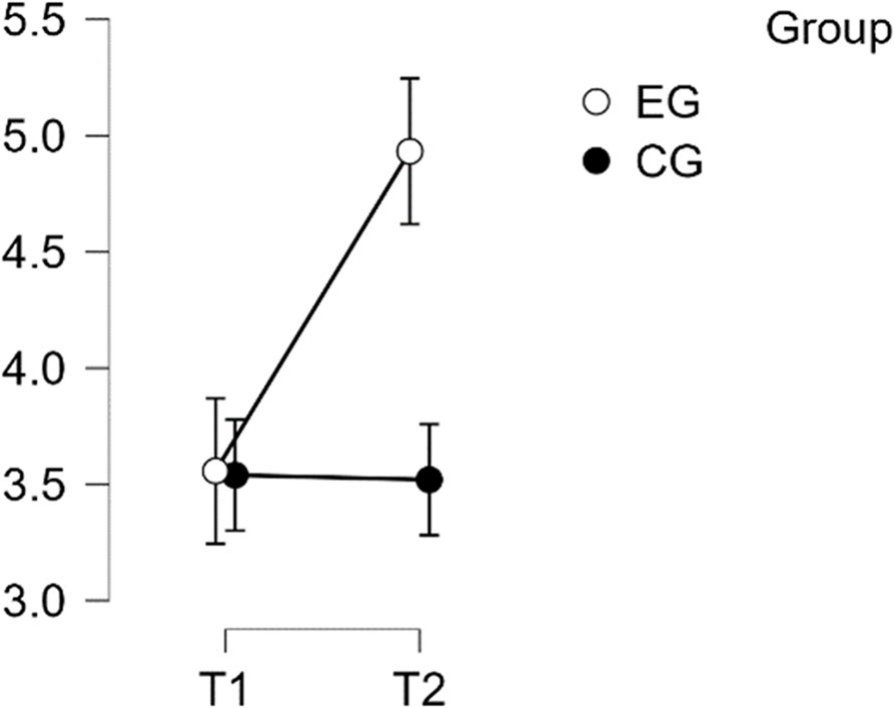
Estimated marginal means for multi-component gratitude measure subscale behavioral shortcoming

Thus, Hypothesis H2 was confirmed regarding the BS subscale (see Fig. 10).

#### MCGM – RNB subscale: rituals/noticing benefits

**Fig. 11:**
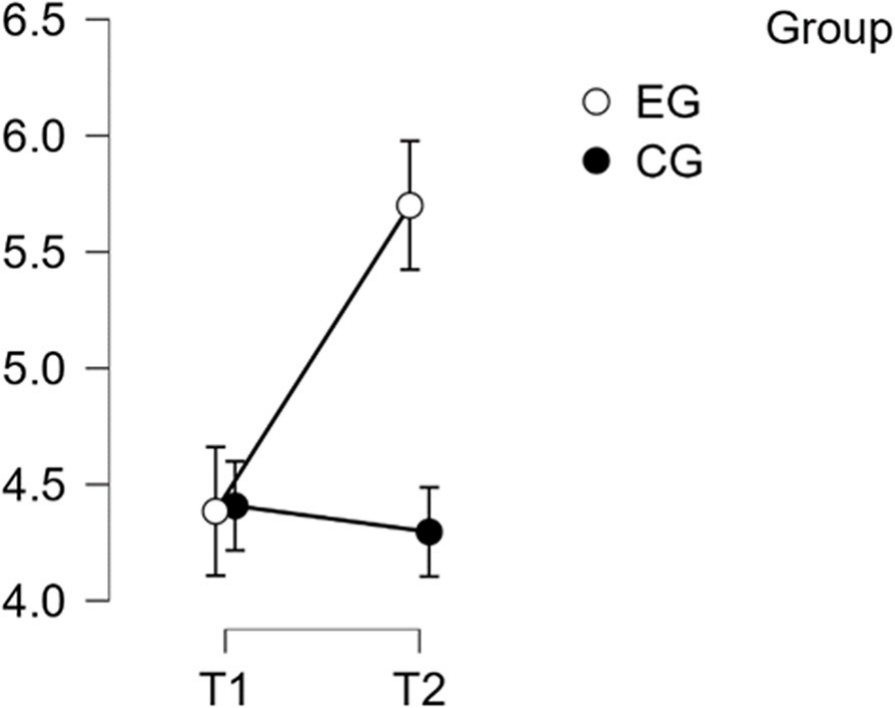
Estimated marginal means for Multi-Component Gratitude Measure Subscale Rituals/Noticing benefits

The analysis showed that there was a normal distribution and equal variance among the groups. The statistical significance of the time * group interaction was established on the RNB subscale, with F(1:49) = 37.667, *p* < 0.001, η^2^p = 0.435. The implicit differences between the EG and the CG were subsequently confirmed through post-hoc tests, with t(51) = 5.054, *p* < 0.001. The intervention resulted in a significant increase in scores from T1 to T2 in the EG, t(26) = −8.078, *p* < 0.001, with a mean difference (MD) of −1.315 points, 95% CI [−1.763, −0.868], and a large effect size (d = −1.326) (refer to Table 12), confirming Hypotheses H2 regarding the subscale RNB (refer to Fig. 11).

**Table 12:** Post hoc test MCGM – RNB subscale

Post Hoc Comparisons—Group ✻ RM Factor 1										
			**95% CI for Mean Difference**					**95% CI for Cohen's d**		
		**Mean Difference**	**Lower**	**Upper**	**SE**	**t**	**Cohen's d**	**Lower**	**Upper**	**p**_**tukey**_
EG, T1	CG, T1	−0.023	−0.778	0.731	0.278	−0.084	−0.024	−0.785	0.737	1.000
	EG, T2	−1.315	−1.763	−0.868	0.163	−8.078	−1.326	−1.902	−0.751	<.001
	CG, T2	0.089	−0.666	0.843	0.278	0.319	0.089	−0.672	0.851	0.989
CG, T1	EG, T2	−1.292	−2.047	−0.537	0.278	−4.651	−1.303	−2.144	−0.462	<.001
	CG, T2	0.112	−0.345	0.569	0.166	0.674	0.113	−0.343	0.569	0.906
EG, T2	CG, T2	1.404	0.649	2.159	0.278	5.054	1.416	0.561	2.270	<.001

*P*-value and confidence intervals adjusted for comparing a family of 6 estimates (confidence intervals corrected using the bonferroni method)

#### MCGM – EOG subscale: expression of gratitude

The analysis showed a violation of normal distribution and confirmation of homogeneity of variance. The statistical significance of the interaction between time and group on the EOG subscale was F(1:49) = 4.040, *p* = 0.050, η^2^p = 0.076. However, post-hoc tests did not confirm any differences between the EG and the CG (t(51) = 2.491, *p* = 0.069). Additionally, the intervention did not result in a significant increase in scores from T1 to T2 in the EG (t(26) = −1.560, *p* = 0.410). Therefore, Hypothesis H2 was not confirmed regarding EOG (see Table 13).

**Table 13:** Post hoc test MCGM – EOG subscale

Post Hoc Comparisons—Group ✻ RM Factor 1										
			**95% CI for Mean Difference**					**95% CI for Cohen's d**		
		**Mean Difference**	**Lower**	**Upper**	**SE**	**t**	**Cohen's d**	**Lower**	**Upper**	**p**_**tukey**_
EG, T1	CG, T1	0.113	−0.510	0.736	0.230	0.492	0.138	−0.622	0.897	0.961
	EG, T2	−0.250	−0.691	0.191	0.160	−1.560	−0.304	−0.839	0.230	0.410
	CG, T2	0.323	−0.300	0.946	0.230	1.404	0.393	−0.373	1.159	0.500
CG, T1	EG, T2	−0.363	−0.986	0.260	0.230	−1.578	−0.442	−1.210	0.326	0.397
	CG, T2	0.210	−0.239	0.659	0.163	1.285	0.256	−0.287	0.799	0.577
EG, T2	CG, T2	0.573	−0.050	1.196	0.230	2.491	0.698	−0.084	1.480	0.069

#### MCGM – AOG subscale: attitude of gratitude

Upon analysis, it was found that normality of distribution and homogeneity of variance were violated. The statistical insignificance of the interaction between time and group on the sub-scale AOG (F(1:49) = 3.979, *p* = 0.052) implies that hypothesis H2 was not confirmed in regards to AOG.

### Results of test for replication – correlation of compassion and gratitude

**Fig. 12:**
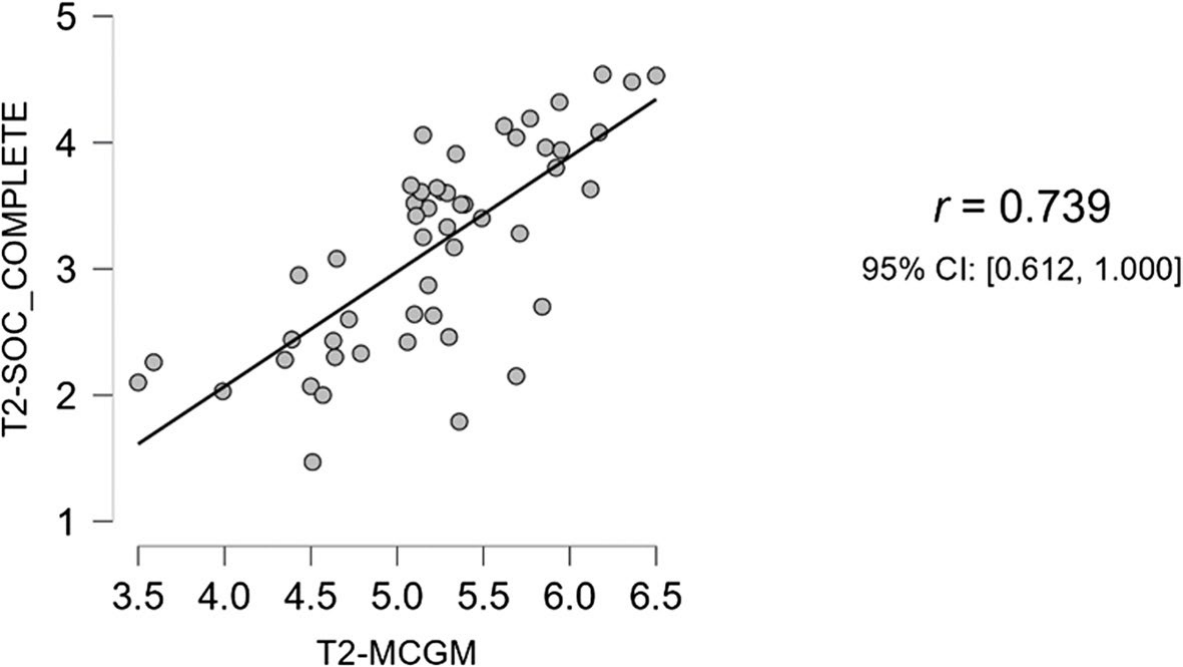
Correlation between self-compassion scale and multi-component gratitude measure scale complete

In addition to the two main hypotheses, we analysed the correlation between the two scales. The Shapiro–Wilk test for bivariate variability suggested normal distribution. The SCS and MCGM were found to be highly positively correlated, with r(49) = 0.74, *p* < 0.001 (refer to Table 14 and Fig. 12).

**Table 14:** Pearson’s correlation between self-compassion scale and multi-component gratitude measure scale complete

Pearson's Correlations					
	n	>Pearson's r	*p*	Lower 95% CI	Upper 95% CI
T2-MCGM	- T2-SOC_COMPLETE	51 0.739	*** <.001	0.612	1.000

All tests one-tailed, for positive correlation. * *p* <.05, ** *p* <.01, *** *p* <.001, one-tailed

## Discussion

The objective of this study was to examine the influence of a four-week training programme on participants'levels of compassion and gratitude, which are crucial elements of women's mental health. The findings indicate that the integration of compassion and gratitude in an easily accessible online intervention can result in significant positive outcomes, underscoring the potential of online interventions as a cost-effective approach to enhance mental health. Prior research has substantiated the efficacy of online training programs analogous to the one that was evaluated here [50]. The hypothesis that this format could enhance levels of compassion and gratitude is consistent with the theoretical proposition that these positive psychological elements are linked to improved mental health outcomes [18–23]. The combination of these two approaches has yielded promising results [22]. The current study revealed a significant enhancement in primary compassion and gratitude constructs within the experimental group (EG) in comparison to the control group (CG). These findings corroborate those of previous studies indicating that compassion and gratitude can be taught and learned [27, 31–35].

During the four-week intervention involving daily metta meditation, all six subscales of the SCS) demonstrated improvement, as did three subscales of the MCGM. However, Feelings of Gratitude and Behavioral Shortcomings exhibited no significant change. The study indicated that gratitude journaling may not be a sufficient standalone intervention and recommended the incorporation of prompts for actively expressing gratitude in future interventions. The group designated for the wait-list control experienced a decline in several measures, with no external factors that could be identified as a clear explanation for this decline. The significant correlation between the compassion and gratitude scales indicates that enhancing one can benefit the other, thereby supporting a practical and adaptable approach to improving women's psychological well-being through positive psychology. The study demonstrated that comparable benefits to those achieved by longer interventions could be attained in a shorter and more cost-effective timeframe.

As the study population was exclusively female, the findings are particularly pertinent to this demographic. The experience of heightened stress is a common phenomenon among women, who frequently encounter challenges in balancing the demands of work, family, and societal expectations [8]. This emphasises the importance of targeted interventions, such as self-compassion and gratitude practices, which can enhance resilience and self-worth [35, 72]. The observed improvements in self-compassion and gratitude indicate that these practices may serve to empower women by fostering a greater sense of self-worth and emotional well-being. This is evident in the definition of self-compassion, which encompasses treating oneself with kindness and understanding in the context of failures and challenges. This approach has the potential to mitigate the impact of self-criticism and foster psychological resilience [35, 73]. Similarly, the core of gratitude practices entails the capacity to shift focus away from negative experiences and foster a more positive outlook on life [40, 54]. Nevertheless, while these practices demonstrate promise, it is imperative to consider the potential limitations and individual differences in responsiveness to these interventions.

The online format of the intervention renders it particularly accessible for women who may have limited time and resources to participate in traditional in-person programmes. It can be challenging for women, particularly those with caregiving responsibilities or demanding jobs, to attend regular therapy sessions or workshops [74]. The flexibility afforded by online interventions allows participants to engage with the material at a time and pace that suits them. Nevertheless, it is essential to recognise potential obstacles, including digital literacy, internet access and the capacity to sustain engagement in an online environment [75]. It would be beneficial for future studies to investigate methods of improving the user experience and retention rates of those participating in online interventions.

While the study demonstrates significant improvements in self-compassion and gratitude, it is essential to undertake a critical evaluation of the study design and consider areas for further research. The exclusive focus on women limits the generalisability of the findings to other genders. Given that females typically report higher self-judgement scores on the SCS compared to males, the results of a study using the SCS with only female participants have specific implications. The findings reflect gender-specific trends and may not be generalisable to males or a mixed-gender population. The higher self-judgement scores typically reported by females may contribute to a greater visibility of the results, particularly in relation to self-judgement. It is therefore possible that the scores may differ in a more gender-diverse sample. Notwithstanding these considerations, the deliberate focus on female participants offers invaluable insights into self-compassion among women, which may be shaped by societal and cultural influences. It is recommended that future studies continue to explore these gender-specific trends. Furthermore, the use of self-reported measures may be susceptible to bias in gereral, as participants may overestimate their improvements due to social desirability or placebo effects [76]. The incorporation of objective measures and longer follow-up periods would facilitate a more comprehensive understanding of the intervention's effectiveness.

Moreover, the study indicates that gratitude journaling may not be a sufficient standalone practice, suggesting the integration of prompts for active gratitude expression. This emphasises the necessity for future interventions to experiment with diverse formats and combinations of practices in order to ascertain the most efficacious strategies. Furthermore, it would be beneficial to investigate the impact of facilitator support and peer interactions on the efficacy of online interventions.

A critical analysis of the demographic data collected reveals that it is subject to a number of limitations. The following variables were considered: gender, age, marital status, experience with gratitude and compassion, and educational level. Nevertheless, the inclusion of further data would have facilitated a more comprehensive understanding of the participants. It would be beneficial for future studies to consider the impact of socioeconomic status on mental health and access to resources, as these factors have been demonstrated to exert a significant influence [77]. An individual's cultural background, including ethnicity, nationality, and cultural beliefs, has been demonstrated to impact their perceptions of and responses to stress, gratitude, and compassion practices [78]. This factor might also be considered in future studies. Moreover, future studies on compassion and gratitude may consider the level of social support from family, friends, and community networks, as this has been demonstrated to have a significant impact on mental health and the effectiveness of interventions [79].

The collation of these supplementary demographic data points will facilitate a more comprehensive analysis of the manner in which different factors interact with the intervention's efficacy in the future. This would facilitate the adaptation of future interventions to better align with the diverse needs of the population, thus improving the efficacy of the interventions.

The absence of long-term follow-up data constrains our comprehension of the training program's lasting impacts. We have reason to believe that a long-term effect is realistic, due to results from other online interventions using the same format in terms of length, media, and trainer [47]. The study aimed to determine whether a self-directed online program led to a significant increase in compassion and gratitude. With this confirmed, future research should build on this goal and include a follow-up study. Additionally, the study sample was recruited solely from the Instagram platform, which may limit sample diversity. For example, only three of the 56 female participants had children, which may restrict the generalizability of the findings to wider populations. Future research should aim to replicate these results in more diverse samples and explore potential moderating variables that could impact the outcomes of interventions. The primary focus of this study was to examine the impact of an online self-directed intervention based on compassion and gratitude training. Further research should also focus on the effects of compassion and gratitude compared to other mindfulness techniques. This can be achieved by setting up an active CG.

In conclusion, the findings of this study highlight the potential of compassion and gratitude practices to enhance the mental health of women, particularly through accessible online interventions. Although the results are encouraging, a critical analysis indicates the need for further research to address limitations and optimise intervention strategies. The adaptation of mental health programmes to the specific requirements and circumstances of women can facilitate their ability to cope with stress and enhance emotional resilience, which in turn contributes to more favourable mental health outcomes.

## Conclusions

In summary, this study provides valuable insights into the effects of a four-week online self-directed training program that promotes compassion and gratitude, with the aim of supporting women's mental health. The hypothesis was that the EGwould experience significant improvements in predetermined constructs related to compassion and gratitude compared to the CG. Furthermore, evidence suggests a positive correlation between gratitude levels and compassion levels. This implies that individuals who report higher levels of gratitude also tend to report higher levels of self-compassion and compassion towards others, and vice versa. These findings highlight the potential benefits of including low-cost, easily accessible online training interventions in mental health promotion strategies, offering both feasibility and flexibility. The improvements observed in the intervention group suggest that skills related to compassion and gratitude can be developed, which previous research has shown to have a positive impact on well-being. However, this study represents an initial exploration of interventions related to compassion and gratitude, and highlights the need for further extensive research. Future studies could investigate the long-term effects of such interventions, taking into account factors such as cultural differences, social obligations, parenthood, and individual variations. Furthermore, future studies should investigate possible domains where increased levels of compassion and gratitude could improve mental health therapy outcomes. As positive psychology continues to evolve, research like this provides a deeper understanding of how cultivating compassion and gratitude can enhance mental health.

## Data Availability

The datasets used and/or analysed during the current study are available from the corresponding author on reasonable request.
